# Development of a compliant spine mechanism for enhanced humanoid robotics locomotion

**DOI:** 10.1038/s41598-025-32165-w

**Published:** 2025-12-27

**Authors:** Amir R. Ali, Hatem S. Abdullah

**Affiliations:** 1 Mechatronics Engineering Department, Faculty of Engineering and Materials Science (EMS), German University in Cairo (GUC), New Cairo, 11835 Egypt; 2 ARAtronics Laboratory, Mechatronics Engineering Department (MCTR), German University in Cairo (GUC), New Cairo, 11835 Egypt

**Keywords:** Tensegrity, Flexible spine, Humanoid robots, Locomotion, Flexinoid, Bio-inspired design, Compliant mechanism, Engineering, Mathematics and computing

## Abstract

Humanoid robots often employ rigid-trunk designs which limit locomotion capabilities and payload capacity. This paper presents a novel bio-inspired, tensegrity-based flexible spine mechanism designed to address these limitations. The design integrates a modular, multi-segment structure combining rigid struts and flexible TPU cables, creating a compliant, stable, and adaptable spine. We developed a novel dynamic model of this tensegrity-based spine to analyze its motion characteristics, providing detailed insights into its load-bearing capabilities and range of motion. Experimental results, obtained using a novel humanoid robot platform (“Flexinoid”), demonstrate improvements in locomotion performance. Furthermore, the design mitigates non-linear movement challenges, allowing for an enhanced range of flexion to -30°:65° and lateral bending by ± 30°. The experimental results confirm an increase in sensitivity and a decrease in the minimum detectable payload following the onset of motor back-driving, validating the effectiveness of the passive energy storage mechanism during initial loading. This enhancement in performance underscores the potential of this bio-inspired design for applications requiring precise control and high payload capacity. This research presents a novel approach to humanoid robot design, paving the way for more versatile and capable robots in various applications.

## Introduction

**Fig. 1:**
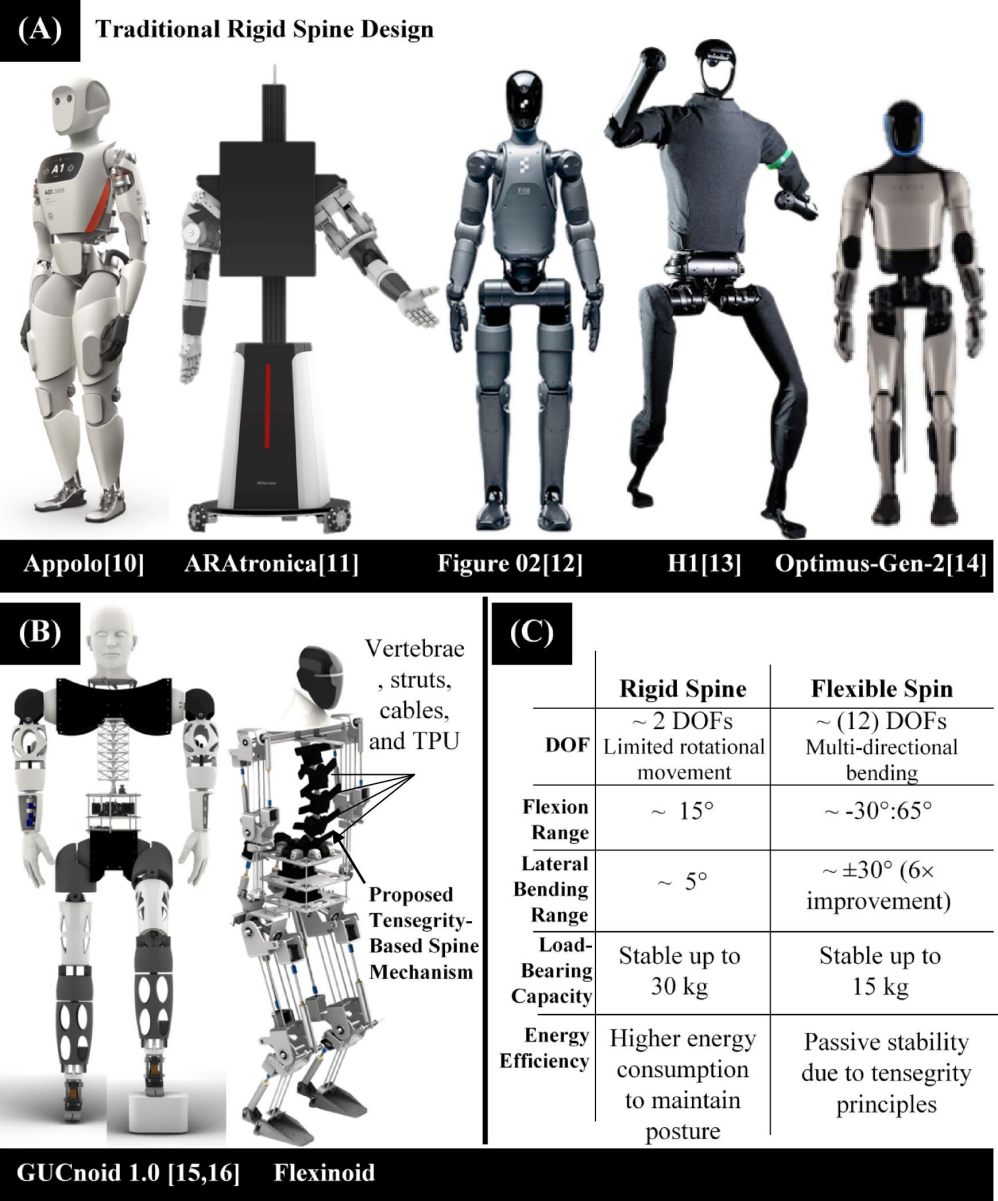
Comparative analysis of humanoid spine designs and the Flexinoid Tensegrity spine. (**A**) Examples of humanoid robots utilizing traditional rigid spine designs, highlighting limitations in flexibility and load-bearing capacity, (**B**). The Flexinoid humanoid robot and a detailed schematic of the tensegrity spine highlighting its components (vertebrae, struts, cables, and TPU), (**C**) Comparison of key design parameters for rigid and tensegrity spine designs, including stability while supporting a ~15 kg payload. The design specifications shown are used to generate the data presented in the Table. (**A**) Traditional Rigid Spine Design: Appolo^10^, ARAtronica^11^, Figure 02^12^, H1^13^, Optimus-Gen-2^14^, (**B**): GUCnoid 1.0^15,16^, Flexinoid

 Humanoid robots hold immense promises for assisting humans in various tasks and environments, offering capabilities beyond those of traditional wheeled robots. Their bipedal locomotion allows them to navigate complex and unstructured environments, mimicking human dexterity and adaptability. However, current humanoid robot designs often incorporate rigid-trunk structures that limit their ability to perform tasks requiring substantial flexibility, such as bending, twisting, or manipulating heavy payloads. These limitations severely restrict their operational range and versatility. Trunk’s degrees of freedom have a contribution to gait cycle and in load distribution in lower section to avoid exceeding actuators limits^1^. Figure 1A illustrates examples of state-of-the-art humanoid robots with rigid spines. The spine reduces the role of the hip and knee in maintaining the stability of the whole body, as the motion in the spine always leads to the motion in the hip^2,3^. This frees up more capacity for the hip and knee to concentrate on locomotion. Existing designs often struggle to maintain stability and efficiency while performing complex maneuvers, especially when handling heavy weights or navigating uneven terrain. Several techniques to maintain whole-body stability against such various perturbations for example ankle strategy and hip strategy^4^. The spinal column has a role in horizontal and vertical acceleration damping of the head, which is essential for a vision system^5^. Attempts to incorporate flexible spines have been made previously, often utilizing continuum robots or complex multi-actuator designs. However, these designs often present drawbacks such as increased complexity, high cost, and limited robustness.

This paper introduces a novel bio-inspired tensegrity-based flexible spine mechanism designed to overcome the limitations of existing rigid-trunk and complex flexible spine designs. Our approach leverages the principles of tensegrity, a structural system characterized by a balance of tensile and compressive forces, along with a modular multi-segment design. This innovative design incorporates lightweight yet robust structure, specifically rigid struts for compression resistance and flexible TPU (Thermoplastic Polyurethane) cables for tension management, creating a stable and adaptable spine mechanism. The modular design facilitates easy assembly, maintenance, and potential future upgrades. The use of flexible TPU cables also introduces compliance to the system, allowing for effective shock absorption. Figure 1B illustrates the design of the proposed tensegrity-based spine, highlighting its modular construction, rigid struts, and flexible TPU cables.

Humanoids with a movable rigid trunk with actuators concentrated in the hip, makes the trunk a rigid inverted pendulum. The force needed from the actuators can be calculated from a rigid cantilever problem in a scenario where the payload to be lifted is in front of the robot. The force is extremely high and challenging to generate by a typical actuator^6^. A hydraulic actuator and servo valve drive^7^ fulfill such loads. Although hydraulic actuators can be customized, they have a high cost and require high machining capabilities^8^. Electric motors with gearboxes^9^. can produce the high torque required, but at the cost of speed reduction, limiting the overall system response.

Figure 1 presents a comparative analysis of humanoid spine designs, showcasing the advantages of the Flexinoid’s novel tensegrity-based spine. Figure 1A illustrates examples of humanoid robots employing traditional rigid spine designs, highlighting their limitations in flexibility and load-bearing capacity. In the human body, spine motion is faster than the motion of the hip^2,3^. The spine’s main target is to fine tune the line of gravity of the upper trunk distressing bipedal mechanism to concentrate on locomotion^5^.

In contrast, Fig. 1B presents the Flexinoid robot with its tensegrity spine, clearly showing its modular multi-segment construction, utilizing rigid struts and flexible TPU cables. This design incorporates an increase in Degrees of Freedom (DOF) compared to the traditional rigid spine designs, as shown in Fig. 1C. The expanded table in Fig. 1C details these improvements in DOFs, flexion range, lateral bending range, load-bearing capacity, and energy efficiency, demonstrating the enhancements achievable using a tensegrity spine design. The Flexinoid’s tensegrity spine requires only 2 motors for achieving multi-directional movement, compared to up to + 20 motors required in its peers humanoids with flexible spine. This improved design leads to a major reduction in energy use compared to rigid spine robots as the proposed spine design has an intrinsic stability which allow the Flexinoid to be stable and handle impulsive perturbations during standing posture. This data illustrates the performance enhancements due to the proposed tensegrity-based design. This paper introduces and evaluates a tensegrity-based, human-scale robotic spine as a load-bearing and passively compliant trunk segment. We report segment-level intrinsic passive stability, shock attenuation, and load redistribution and show that these properties reduce the burden on actuators required for trunk manipulation. Whole-system dynamic performance (full humanoid locomotion and disturbance rejection) is outside the scope of this study.

## Literature survey

The design of flexible and adaptable spines for humanoid robots presents challenges. Existing designs struggle to achieve the necessary balance between flexibility, stability, load-bearing capacity, and energy efficiency. Rigid-spine designs, while simple, severely limit the robot’s dexterity and ability to perform tasks requiring bending or twisting^1^. Attempts to create more flexible spines have used several different approaches, each with its own set of limitations.

Bio-inspired spines depend on multi-degree-of-freedom joints, soft tissues, and variable stiffness joints and structures. The musculoskeletal system consists of bones, cartilage, tendons, ligaments, and muscles. The bones role is to handle loads throughout the whole skeleton. Ligaments are passive elements that guide motion and enhance intrinsic stability of each joint. Cartilage acts as shock absorbance inside the joints. Musculoskeletal robots are inspired by living organisms; a lot of studies have emerged in this field, such as mechanically compliant modules^8,17^ that replicate the effect of cartilage between joints and muscle elasticity. Musculoskeletal robots main objective is to make humanoids gain Bio-inspired embodied intelligence as medical research platforms^18^ or by taking advantage of hardware intelligence for the sake of high performance and physical resilience at low-level computational capabilities^19^. Bio-inspired spines use continuum robots such as Kenta^20,21^, Kenzoh^22^, Kenshiro, and Kengoro^23^. Spherical joints used in continuum robots can only handle compressive loads; therefore, the structure is prone to collapse if a tensile load hits the system. Kenta needs 40 motors with tension sensors to control the spine, resulting in complex control to prevent intervention between muscles and out-of-control situations. By changing the actuation from linear muscle to planar in the Kenzoh Humanoid, the number of motors is reduced, but the designed module needs continuous maintenance so that pullies transmit motion through the mechanism properly. Kenshiro and Kengoro are good examples of anthropometrically correct humanoid robots with a combination of planar and linear muscles. Kenshiro has 64 muscles, and Kengoro has 174 muscles. Such robots need extensive analysis and computation power to have synchronization between all these muscles to follow, for example, the gait cycle. Therefore, it is essential to adopt a methodology that goes beyond simply duplicating the actual musculoskeletal system as just replication will be very challenging. Its important to comprehend and use bio-inspired embodied intelligence, which results from the redundancy of the complex musculoskeletal system. In this study, tensegrity principles are integrated to the spine structure to add intrinsic stability to the spine.

Tensegrity structures, characterized by a balance of tensile and compressive forces, have emerged as a promising approach for creating lightweight yet robust and adaptable compliant mechanisms^24^. The inherent stability of tensegrity structures makes them particularly suitable for creating flexible spines in humanoid robots^25^, potentially reducing the complexity and cost associated with multi-actuator designs while maintaining structural integrity. Moreover, tensegrity structures offer inherent shock absorption and energy efficiency^19^, further improving their suitability for robotics applications.

This research builds upon the existing literature by introducing a novel tensegrity-based flexible spine mechanism for humanoid robots. Our design integrates a modular, multi-segment structure that addresses some of the limitations highlighted above. We have also developed a novel dynamic model to analyze the system’s behavior, providing detailed insights into its performance characteristics. The following sections describe this innovative design, its dynamic modeling, and its experimental validation.

**Fig. 2:**
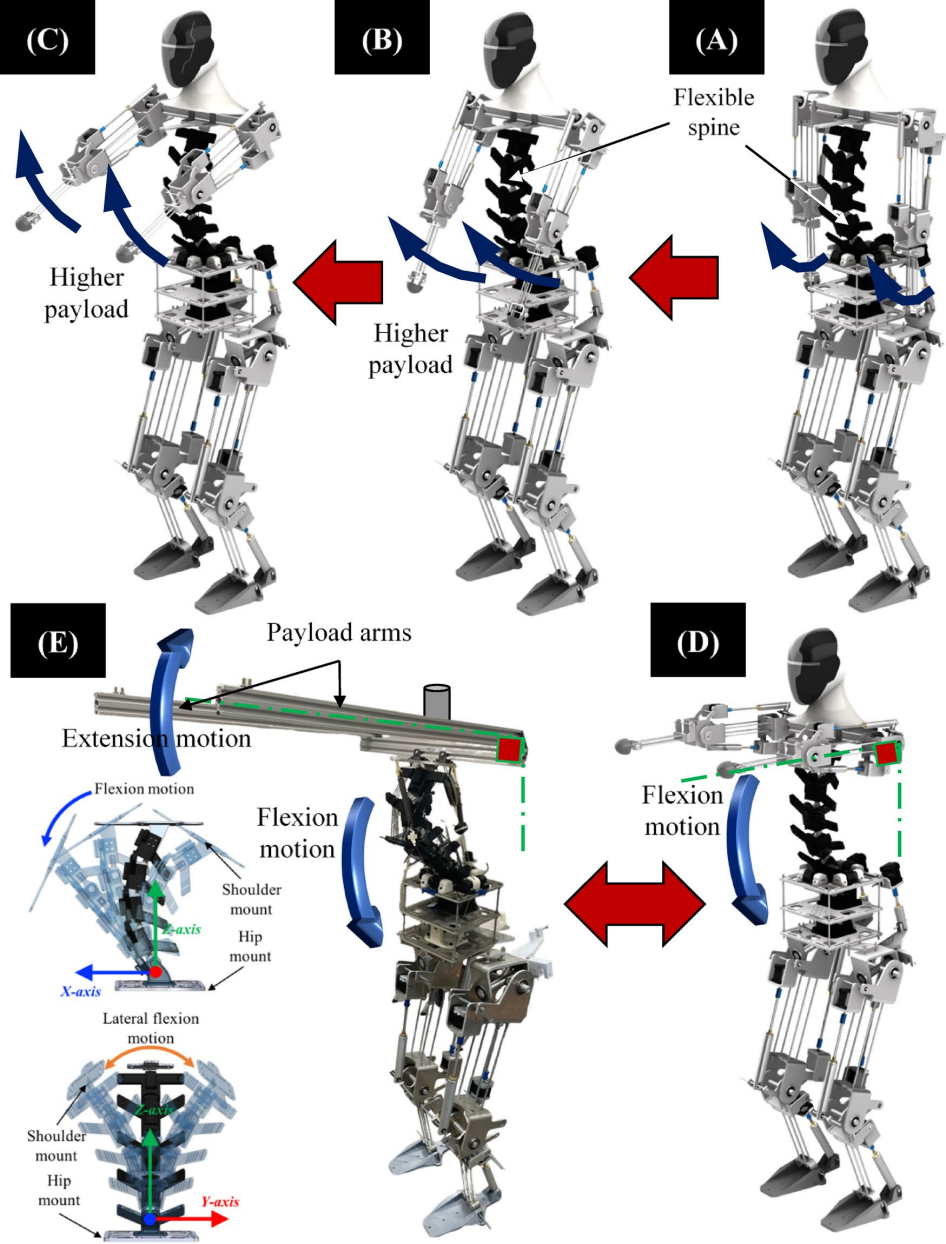
Flexinoid Spine Functionality and Payload Interaction. (**A**–**C**) Sequence of payload increase by increasing the arm of moment of the added weights in sagittal plane., (**D**) Spine passive reaction to payload increase (**E**) Spine response to a payload using a rigid arm. Note: For clarity, X, Y, Z axes are consistently colored Blue, Red, and Green, respectively, and motion arrows around Y are standardized to blue.

Figure 2 presents a series of images illustrating the dynamic movement capabilities of the research platform, the Flexinoid with the integrated tensegrity spine mechanism. Manipulating payload using the robot arm shows the capabilities of the flexible spine and its interaction with an external payload. The figure is divided into five sub-Figures 2 A-2E, showcasing different stages of payload addition and configurations. In Figs. 2 A, 2B, and 2C Blue curved arrows present a sequence of sagittal plane payload addition by increasing the arm of moment of the added weights. The robot’s spine displays flexion (bending forward) and extension (returning to an upright position) Fig. 2D and E. The blue arrow in Fig. 2D and E show the direction of the spine movement during loading as a passive reaction to payload increase for the robot maintains an upright posture throughout these movements, suggesting the importance of spine design for balance. Figure 2D shows the robot holding a payload attached to its arm. This configuration demonstrates the spine’s role in maintaining balance while supporting an external load. Figure 2E shows a modified configuration where the robot’s arm has been replaced with a dedicated payload arm (shown as a bar with a load at the end), allowing for a focused examination of the spine’s mechanical response to external loads. The labels “Flexion motion” and “Extension motion” clarify the type of movement shown in each subfigure. This simplification of replacing arms mechanism with two rigid V-slots enables the isolation and evaluation of the spine’s behavior under different load conditions, demonstrating its capacity for managing various payloads. The illustrations of the flexion and extension motions in this setup further highlight the spine’s ability to support the load while performing extension motion. In summary, Fig. 2 demonstrates the robot’s ability for multi-planar movements and its ability to withstand external payloads depending on structure intrinsic stability. The figure emphasizes how flexible spine’s react when the arms move while picking a load, the sequence of images suggests a dynamic experiment evaluating the spine mechanism’s reaction to load-bearing. The removal of the arm in Fig. 2E is particularly important because the rest of the paper focuses on the spine’s mechanics.

**Fig. 3:**
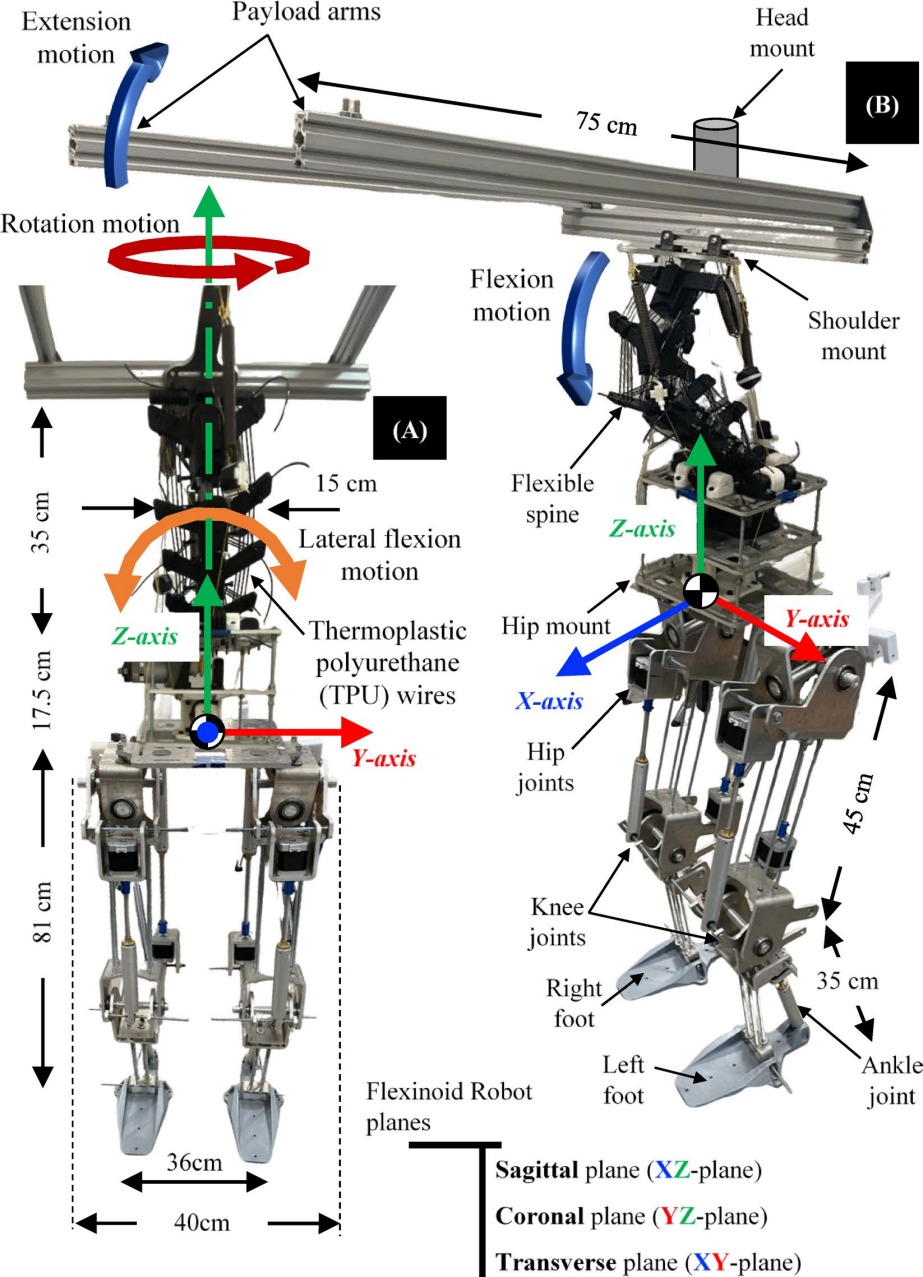
Flexinoid humanoid robot featuring a flexible spine. (**A**) Front view of the robot highlighting key dimensions and the location of the flexible spine, (**B**) range of motion provided by the flexible spine and anatomical planes. Note: For clarity, X, Y, Z axes are consistently colored Blue, Red, and Green, respectively, motion arrows around Y are standardized to blue, motion arrows around X are standardized to orange, and motion arrows around Z are standardized to dark red.

The Flexinoid’s spinal motion is divided into two key components as clear in Fig. 3 to mimic the human spine’s functionality. Flexion/extension (pitch) and lateral flexion (roll) in the sagittal and coronal planes, respectively, are controlled via actuators in the lumbar region—replicating the human spine’s primary functions. Yaw motion (transverse plane rotation) is independently managed via a revolute joint between the shoulder mount and upper body. This modular approach enhances both biomimicry and the robot’s dexterity.

The Flexinoid spine design integrates two established axial column principles: continuum mechanics and tensegrity. The continuum aspect addresses the spine’s role in bearing compressive and tensile loads, while the tensegrity structure enhances intrinsic stability and transmits motion throughout the spine segments. Utilizing rod-end spherical bearings at each intervertebral joint provides a self-lubricating, durable, and easily maintainable spherical joint. Tendon-driven actuation, augmented by a series of elastic elements, efficiently controls motion and enhances payload capacity. This system leverages gravitational potential energy during downward motion, releasing it during upward movements to assist the actuators and handle loads beyond the motors’ individual capabilities. The resulting design exhibits both stiffness for precise movements and compliance for shock absorption, contributing to the intrinsic stability of the Flexinoid’s spine. Here, intrinsic stability refers to the spine’s ability to return to its original equilibrium position after being subjected to a perturbation, reflecting the passive restorative forces within the compliant mechanism. Further quantitative validation of this intrinsic stability is presented in the Results section.

The Flexinoid humanoid robot, as depicted in Fig. 3 incorporates a novel flexible spine design inspired by the human musculoskeletal system. While the human spine’s complex S-curve distributes loads via a balance of compression and tension, the Flexinoid simplifies this to the lumbar region’s primary pitch and roll motions—mirroring the human lumbar spine’s primary function—with minimal yaw, consistent with the observation that yaw primarily originates in the human thoracic region^5,26,27^. So the yaw motion then is added at the chest reign.

## Materials and methods

**Fig. 4:**
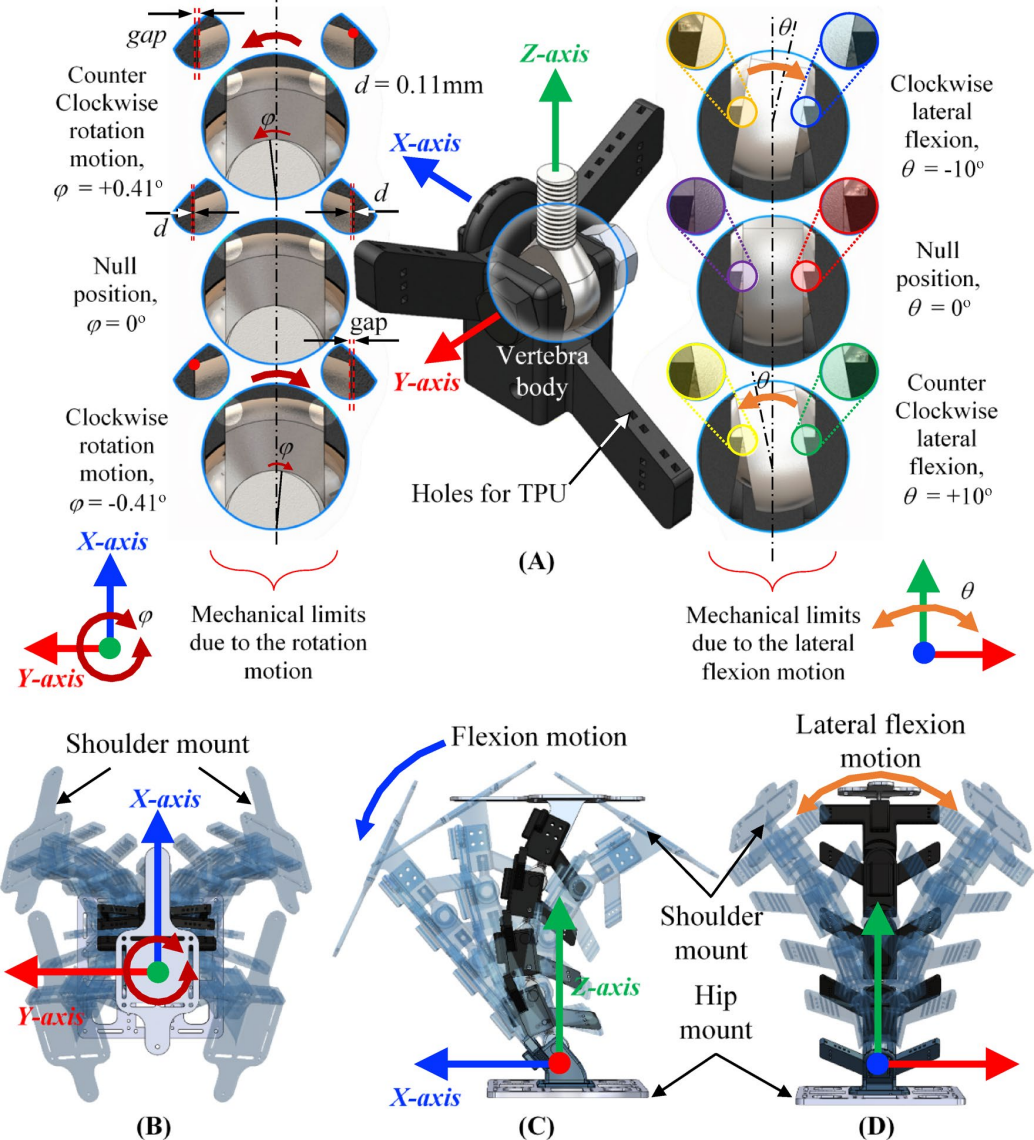
Design and mechanics of the Flexinoid vertebra and spine. (**A**) Detailed view of a single vertebra highlighting strut integration, TPU cable holes, and the mechanical limits due to the lateral flexion motion and rotation motion, (**B**) illustration of mechanical limits restricting rotation and ensuring distributed motion, (**C**) flexion motion of the spine demonstrating the range of movement, (**D**) lateral flexion motion of the spine. Note: For clarity, X, Y, Z axes are consistently colored Blue, Red, and Green, respectively, motion arrows around Y are standardized to blue, motion arrows around X are standardized to orange, and motion arrows around Z are standardized to dark red.

This research utilizes the Flexinoid platform, a novel humanoid robot specifically designed for investigating flexible spine mechanisms. The Flexinoid features a modular design, enabling easy assembly and maintenance, and incorporates an actuation system with integrated sensors. The Flexinoid’s tensegrity-based spine comprises five vertebrae, each incorporating rigid PETG 3d printed body including tensegrity struts (50 mm length) and flexible TPU cables (1.75 mm diameter). The struts are precisely integrated within each vertebra, forming a tensegrity network, and the TPU cables are routed through carefully designed channels, as shown in Fig. 4A. The materials were chosen for their high strength-to-weight ratio, durability, and flexibility. Each vertebra is designed with three degrees of freedom (DOFs): flexion (25°), lateral bending (± 10°), and rotation (± 0.41°), enabling a wide range of motion. The total number of DOFs in the spine is 12. Figure 4 shows a view for each vertebra of the Flexinoid spine and the relationship between individual vertebrae and its components, while Fig. 4B and D shows the extreme positions of the spine guided by the mechanical limits responsible for restricting rotation and lateral flexion motion. The spine S-curve and the angels of vertebrae are inspired by the real human spine curvature^28^. The proposed spine was designed to fit manufacturing capabilities and Flexinoid body ratio requirements.

### Mechanical limits and the range of motions

The human spine achieves stability through a combination of passive ligamentous support and active muscular control^5^. Ligaments are passive elements that play a crucial role in force transmission throughout the vertebral column (spinal coupling)^27^. Inspired by this complex system, each vertebra in Flexinoid spine has holes for the TPU to incorporate a tensegrity structure as in Fig. 4A comprised of rigid struts (attached to each vertebra) and flexible cables embedded within the struts (holes in the struts to connect the TPU). This design provides both structural integrity and compliance, facilitating efficient force transmission along the spine. In the human spine, ligaments and apophyseal joints act as mechanical limits to prevent excessive intervertebral motion and protect the spinal cord^5,27^. The allowable elongation in spine ligaments is less than 35% and if this limit is exceeded, an injury occurs^5^. The Flexinoid design mimics this function using mechanical limits integrated into each vertebra illustrated in Fig. 4A, restricting lateral, transverse, and flexion movements. This distributed motion strategy is crucial for maintaining spinal stability and longevity, preventing excessive stress on any single segment. Limiting the range of motion in each vertebra is particularly important during flexion, preventing hyperextension beyond the natural range and ensuring that the actuation force line doesn’t exceed any pivot of rotation^22^, thereby avoiding destabilizing forces. Replicating the human spine’s complex intervertebral articulation, which allows for multi-axial movement via intervertebral discs, presents design challenges for robotic systems. The Flexinoid addresses this complexity by employing a simplified model of spherical joints for intervertebral articulation as seen in Fig. 4, reducing mechanical complexity while maintaining sufficient range of motion. Although human vertebrae exhibit limited rotation and translation^5,27^, the Flexinoid design subtly lowers each vertebra’s pivot point, resulting in a combination of rotation and translation in the proximal vertebra. This approach, with its guided intervertebral motion, yields a mechanically stable and predictable movement pattern for the spine, further enhanced by the incorporation of mechanical limits (Fig. 4A and C) to maintain the pivot point within a safe range of motion. This predictability in motion is a key aspect of its intrinsic stability, where the spine consistently follows defined kinematic trajectories under specified conditions. To prevent instability and failure, the Flexinoid spine incorporates mechanical limits and guides integrated into each vertebra as clear in Fig. 4A. This design mimics the human spine’s use of articulated processes^5^, which function similarly to cam and slider mechanisms, to guide and control intervertebral motion, thereby ensuring smooth and controlled movement.

The Flexinoid spine’s design incorporates several key features at the vertebral level that contribute to the overall spinal column’s properties and functionality. The arrangement of struts and TPU cable routes (Fig. 4A) creates a tensegrity network analogous to the human spine’s ligamentous support. Integrated mechanical limits within each vertebra ensure that motion is distributed across the entire spine, preventing excessive movement in any specific vertebra. Integrated mechanical limits constrain both rotational and lateral flexion movements within the Flexinoid spine, ensuring that motion is distributed across multiple vertebrae (Fig. 4A). This prevents any single segment from experiencing excessive stress or movement. Figure 4B shows the boundary of unconstrained motion, illustrating coupled sagittal and lateral plane movements. Figure 4C and D illustrate the range of flexion and lateral flexion, respectively. The mechanical limits prevent hyperextension beyond the natural range of motion during flexion, maintaining the actuation line of force within the pivot of rotation^22^. Each vertebra incorporates a rod-end spherical bearing, which provides a low-friction, multi-axial joint. Lateral rotation (around the X-axis) is mechanically limited (*θ* to ± 10°), while transverse rotation is minimized and instead actuated at the chest. The bearing consists of a ball and body; the body is constrained by a planar mechanical limit, creating a controlled rotational movement around the X-axis. However, because the bearing body is offset from the axis of rotation, this constrained motion also includes a translational component. A small gap (*d* = 0.11 mm, Figure. 4A) between the bearing body and the mechanical limit permits limited transverse rotation (around the Z-axis), with a range of motion (*ϕ* of ± 0.41°).

### Tensegrity network and motion constraints

The Flexinoid spine achieves its inherent stability and efficient force transmission through a meticulously designed tensegrity network (Fig. 4A), drawing inspiration from the human spine’s passive ligamentous support and spinal coupling^5,27^. This network is characterized by its continuous tensile elements (TPU cables) and discontinuous compressive elements (rigid struts (50 mm length) embedded within each vertebra, Fig. 4A), which together provide both structural integrity and tunable compliance. Beyond contributing to intrinsic stability, these flexible cables facilitate distributed force transmission throughout the vertebral column. To achieve passive stability, the tensegrity structure’s design was guided by principles of pre-stressed equilibrium. Each vertebral segment integrates four TPU cables (1.75 mm diameter), forming a cross-braced configuration between adjacent vertebrae. These cables are routed through precisely engineered channels within the struts and vertebra body (Fig. 4A), ensuring smooth paths that minimize friction and abrasion during motion. The number of cables (~ four per segment per each strut) was chosen to provide sufficient redundancy for multi-axial stability while minimizing structural complexity and weight.

The crucial aspect of achieving passive stability lies in the initial tensioning of these TPU cables. During assembly, the cables are manually pre-tensioned. This pre-tension creates a stable, custom-made equilibrium orientation for the spine. This means that when the spine is perturbed from this equilibrium, the inherent tensile forces within the tensegrity network generate restorative moments that passively return the system towards its initial, stable configuration, without requiring active motor control to counteract small disturbances. The routing paths (e.g., specific attachment points and lead-in angles) were optimized to ensure that cable tensions generate restoring forces for pitch and lateral flexion, leveraging the compliant properties of the TPU. The flexible TPU was selected for its elastic modulus, which allows for significant elongation under load while providing the necessary restorative force.

Integrated mechanical limits within each vertebra (Fig. 4A) serve a dual purpose where they restrict excessive intervertebral motion, mimicking the protective role of human ligaments and apophyseal joints^5,27^, and they ensure motion distribution across the entire spine. This distributed motion strategy is critical for maintaining long-term stability and preventing localized stress or mechanical failure. These limits constrain lateral (rotation about X-axis) and transverse (rotation about Z-axis) movements to ± 10° and ± 0.41° respectively, while flexion is limited to 25°. Figure 4B illustrates these mechanical limits restricting rotation, ensuring distributed motion across multiple vertebrae. Figure 4C and D further demonstrate the individual ranges of flexion and lateral flexion. Limiting intervertebral flexion is particularly important to prevent hyperextension beyond a straight posture, thereby ensuring that the actuation line of force remains within the pivot of rotation and avoids destabilizing forces^22^.

### Design and actuation for the flexinoid’s spine

**Fig. 5:**
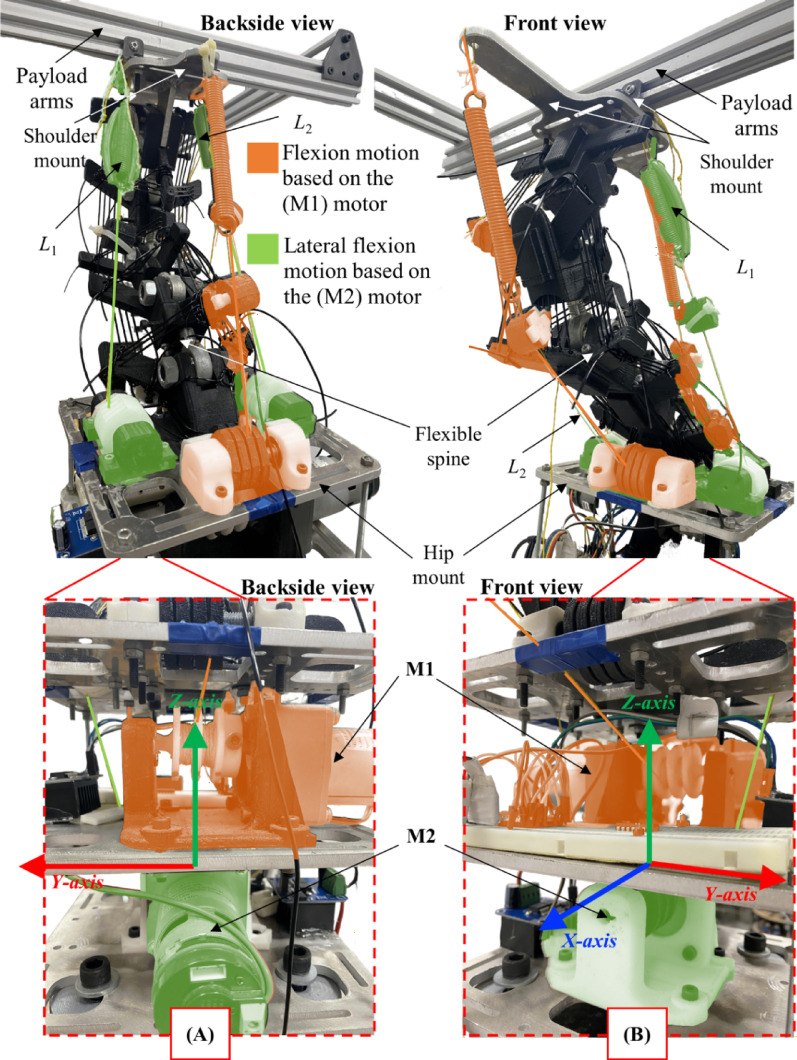
Flexinoid’s spine: Design and Actuation. (**A**) Backside view showing the robot’s structure, including payload arms, shoulder mount, flexible spine, and hip mount; color-coding indicates the actuators responsible for flexion (M1, orange) and lateral flexion (M2, green) movements, (**B**) Front view of the spine mechanism, highlighting the M1 (flexion) and M2 (lateral flexion) motors, and the coordinate system (X, Y, Z axes).

The spine has to main actuated motions; Motion in sagittal plane that bend spine forward (flexion) and backward (extension), and motion in coronal plane that moves the spine laterally to the right and the left. Motor M1 is responsible for the motion (flexion in positive direction and extension in negative direction Fig. 4C) in the sagittal plane. Motor M2 controls lateral motion in the coronal plane (with positive sign to the left and negative to the right Fig. 4D). Each motor drives a pair of cables routed in opposite directions around a dedicated pulley fixed to the motor shaft (see the lower section of the back and front views in Fig. 5). One cable is wrapped clockwise, the other counterclockwise. When the motor rotates, one cable reels in (increasing tension) while the other pays out (decreasing tension). This arrangement produces bidirectional actuation about the target axis without requiring a separate return spring: the cables themselves form a push–pull pair through tension exchange. To ensure clean, reliable winding, each pulley provides two physically separated grooves/slots—one per cable. These independent tracks reduce crossovers or knotting. By separating the cable paths, the windings *cannot* climb over each other, which prevents cable crossover, knotting, or wedging during reversals reducing wear and friction. Separation eliminates cable-on-cable rubbing, lowers abrasion, reduces heat build-up, and extends cable life. Motor 1 loop (highlighted in orange in Fig. 5) is responsible for flexion/extension motion. Motor 2 loop (highlighted in green in Fig. 5) actuates the spine lateral motion (Right/left). In practice, each motor–pulley–cable set is assembled with a small pre-tension in both cables. Pre-tension removes slack, reduces backlash, and ensures that the transition between “pulling” and “releasing” is smooth rather than stepwise. The tension manual tuning mechanism is attached to each cable for fine tuning Fig. 5. Cable routing is planned to avoid abrasive bends and to maintain generous bend radii at guides so the cable fibers are not fatigued prematurely. The rotation of the motor directly translates to intervertebral motion. Mechanical limits and the tensegrity structure translate motion through the spine segments from top to bottom as an analogy to spinal coupling in human spine^27^. The cables act as a tendon-driven actuation system to control spine movement.

Within the cable loops driven by Motor 1 and Motor 2, each cable includes an inline elastic actuator (series spring). Placed in series with the tendon path. The springs in lateral loop L1 and L2 can be seen in Fig. 5. This element introduces a compliance that benefits both mechanics and control. The series spring behaves like a mechanical low-pass filter for force. Sudden load changes (e.g., contact, slip, small impacts) are absorbed as temporary spring deflection rather than transmitted directly to the motor shaft. During flexion (cable reel-in), the spring is loaded and stores elastic energy, and during extension (cable pay-out), the stored energy is released, producing helpful cable tension that assists the motor. Practically, this means the system delivers extra effective torque at the onset of extension, which is the harder part of the cycle: extension works against gravity and payload, whereas flexion generally occurs with the direction of the falling load and therefore requires less effort. During flexion, the series spring stores elastic energy; at the transition into extension, that energy is returned to the tendon, boosting motor torque. This will be addressed again in the passive load bearing experiment.

The current setup employs open-loop control via a joystick, allowing for manual control of the spine’s movement. Closed-loop control will be implemented in future work. Figure 5 presents both front and back views of the robot and highlights key components including the payload arms, shoulder mount, flexible spine, and hip mount. Color-coding of the actuators differentiates between the motors responsible for flexion (M1, orange) and lateral flexion (M2, green). The detailed close-up views highlight the spine mechanism’s layout, illustrating the M1 and M2 motor positions within the coordinate system (X, Y, Z axes). This modularity in design is key to the functionality of the spine.

## Experimental work

The primary experimental validation of the Flexinoid spine mechanism focused on its dynamic response to varying payloads and moment arms. This was conducted using the Flexinoid robot platform and it would be discussed at the end of this section with a dedicated testing apparatus. To isolate and evaluate the spine’s mechanical response to external loads, the robot’s standard arms were replaced with a specialized payload arm system (Fig. 2E). A baseline payload of 1.8 kg, representing the weight of the payload arm system itself, was initially attached. Additional payloads were then incrementally added, ranging from 1 kg up to a maximum of 6 kg, for a total payload (arm + added weight) of 7.8 kg. These experiments were performed at three distinct moment arm lengths, measured from the shoulder joint: d₀=25 cm, d₁=50 cm, and d₂=75 cm. The decision to limit dynamic load testing to 6 kg (for a total of 7.8 kg including the arm) was based on: (1) focusing on the critical transition from passive energy storage to active motor back-driving within this range, and (2) ensuring precise and controlled manipulation within the current capabilities of the active actuation system without risking over-torquing the motors during dynamic operations. High-resolution cameras were positioned to capture the spine’s motion. Reflective markers were affixed to each vertebra for accurate motion tracking. The overall experimental setup, illustrating the robot platform, payload application points, and camera positions, is depicted later in this section. This setup provided a controlled environment for quantitative analysis of spine flexion, load-bearing capability during active manipulation, and intrinsic stability.

**Fig. 6:**
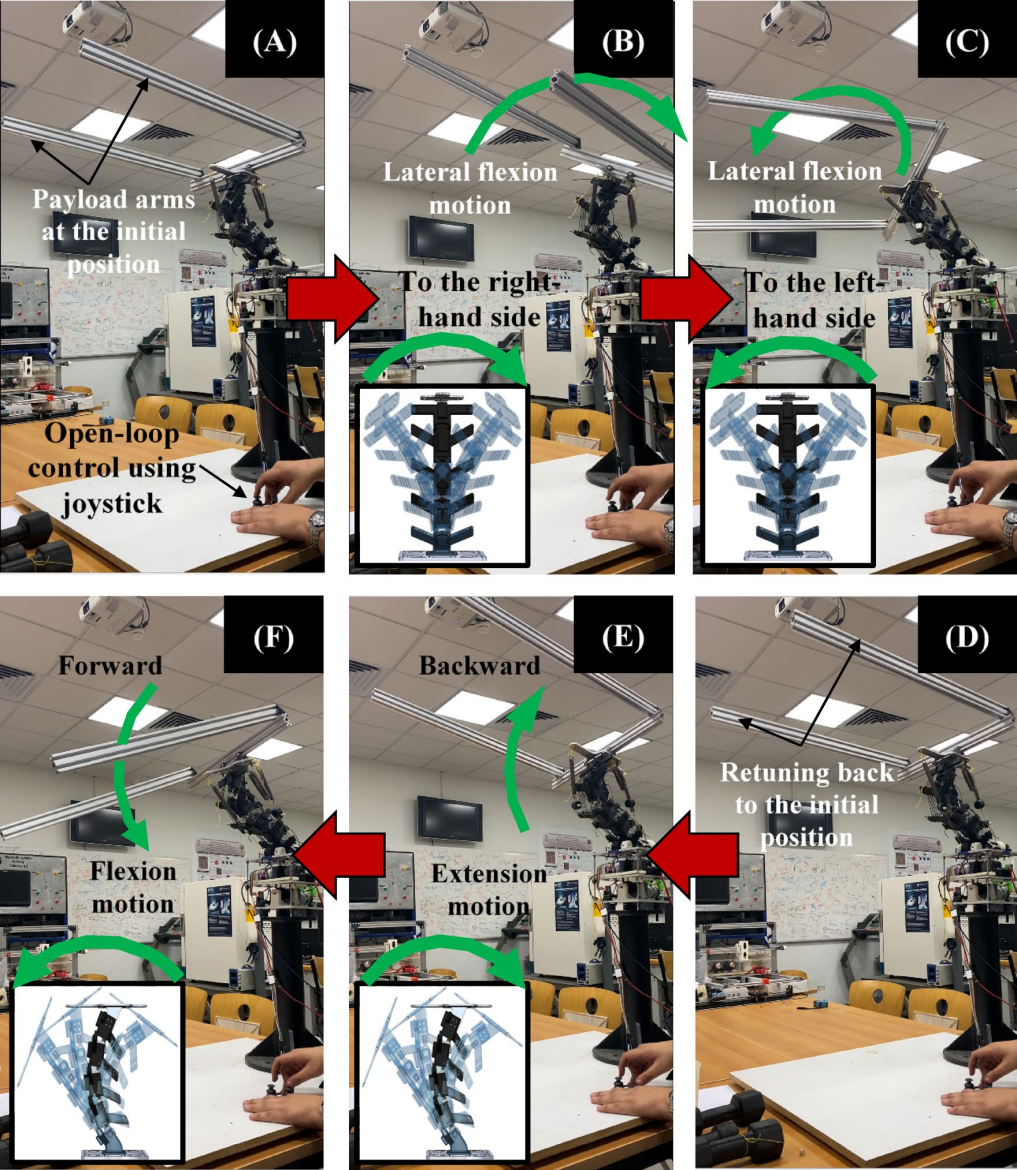
Experimental Demonstration of Flexinoid’s Open-Loop Controllability Using a Joystick with 3 kg payload. (**A**) Initial payload arm position, (**B**) Lateral flexion motion to the right, (**C**) Lateral flexion motion to the left, (**D**) Forward flexion motion, (**E**) Backward extension motion, (**F**) Return to initial position.

Figure 6 presents a time-sequenced demonstration of the Flexinoid trunk actuated open-loop via a handheld joystick—i.e., without sensor feedback or closed-loop compensation. During these trials, a 3 kg rigid arm was mounted at the shoulder interface to emulate an upper-limb payload. The arm’s center of mass was intentionally offset 25 cm lateral to the spinal y-axis, creating a constant bending moment about the trunk. The experiment systematically explores the robot’s range of motion in both the sagittal (forward/backward flexion) and coronal (lateral flexion) planes. The figure is composed of six sub-Fig. 6A and F, each capturing a distinct stage in the experimental process. Figure 6A and B, and 6C illustrate the robot’s lateral flexion motion capabilities. The robot’s arms are initially positioned at a neutral position (Fig. 6A), then flexed to the right (Fig. 6B), and finally to the left (Fig. 6C). Green arrows indicate the direction of lateral flexion motion, and inset images show the corresponding configuration of the spine, demonstrating the spine’s multi-directional bending. Red arrows show the transition between different phases. Figure 6D and E, and 6F show a sequence of sagittal plane movements (forward and backward flexion). Starting from a neutral posture (Fig. 6D), the robot is then controlled to move backward (extension motion, Fig. 6E), forward (flexion motion, Fig. 6F), and finally returns to its neutral state (Fig. 6D). Green arrows show the direction of flexion and extension movements, and the inset images illustrate the spine’s corresponding configuration. Red arrows again indicate the transition between poses. So, Fig. 6 provides a visual record of an experiment demonstrating the Flexinoid robot’s open-loop controllability, showcasing the robot’s range of motion using a joystick and illustrating both lateral and sagittal plane movement capabilities. The clear labeling, arrows, and inset images communicate the experiment’s methodology and results. The use of inset images showing the spine configuration at each stage is particularly effective in illustrating the relationship between control inputs and the robot’s response. The figure highlights the robot’s ability to smoothly and accurately perform flexion and extension movements using an open-loop control method.

**Fig. 7:**
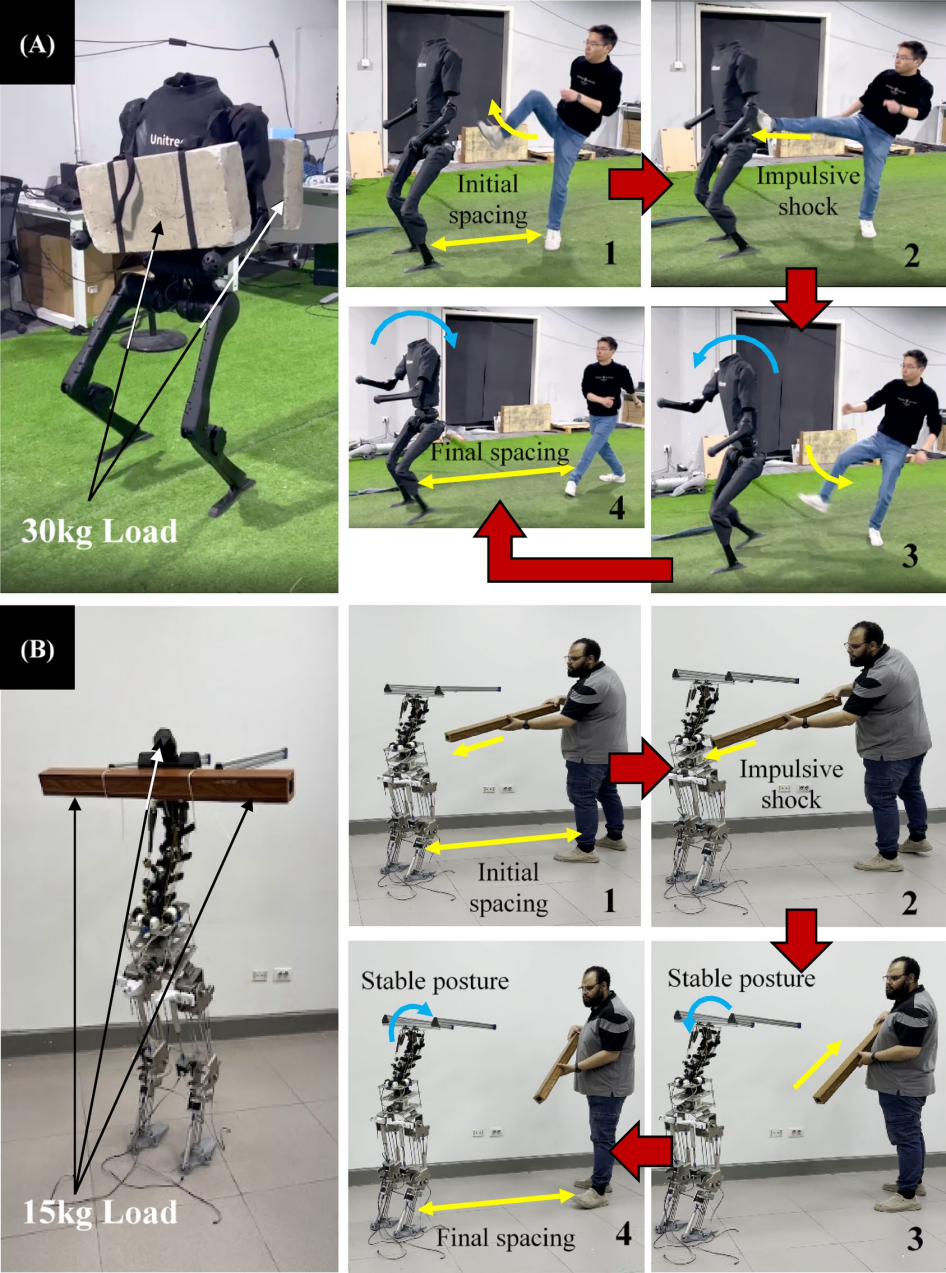
Comparative shock absorption and stability response of Unitree H1 and Flexinoid humanoid robots under impulsive external disturbances. (**A**) H1 robot can support up to a 30 kg load loses balance after hip impact due to its rigid trunk structure^13^. (**B**) Flexinoid, equipped with a tensegrity-based spine can support up to 15 kg passive load, maintains upright posture and stability post-impact, demonstrating effective passive shock absorption and trunk compliance.

Rigid-torso humanoids (e.g., Unitree H1^13^ typically rely on high-torque hip actuators and active control to maintain trunk posture. By contrast, the Flexinoid’s tensegrity-assisted flexible spine exhibits structural passive behavior: with no actuators powered, the trunk passively supported up to 15 kg versus max payload of 30 kg with active (Motors are powered on) 220 N.m hip actuator in Unitree H1 (Under the same trunk fixture)^13^; as seen in (Fig. 7A and B). The spine also permitted active manipulation of a 3 kg payload using 1.89 N·m actuators (Fig. 6). These outcomes are enabled by the spine’s distributed compliance (tensegrity network) and series elasticity in the tendon paths, which together share load, absorb shocks, and reduce instantaneous torque demands at the motor. Our objective here is not to compare whole-system performance, but to show trunk-level passive and assisted behavior of a tensegrity spine under specified boundary conditions (fixture geometry, payload mass, and moment arm). Accordingly, we report a segment-level payload-to-actuator-torque ratio under those conditions. The H1 figures are cited only as design context representative of rigid-torso approaches; a like-for-like system comparison is outside the scope of this study.

**Fig. 8:**
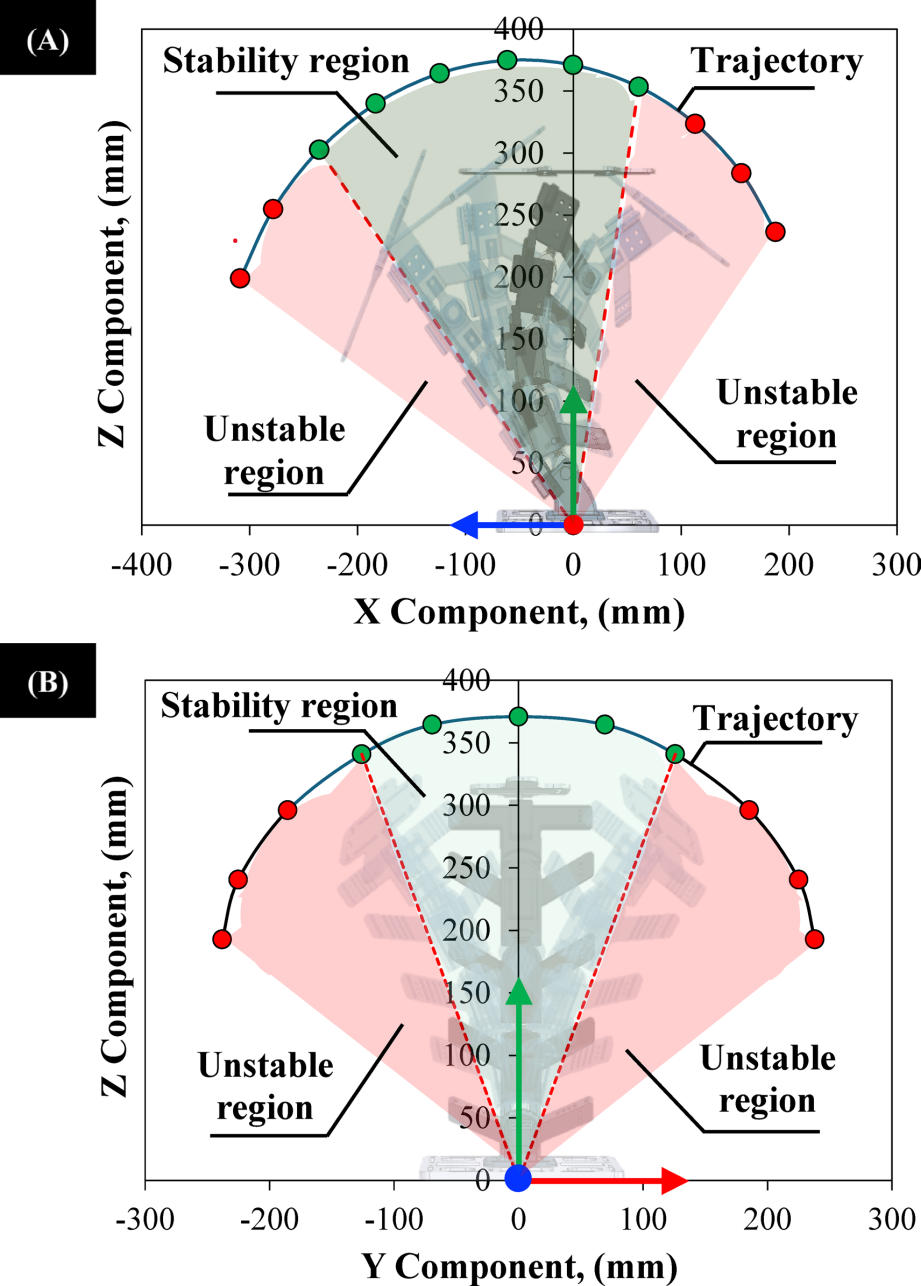
Sability regions and trajectory for the Flexinoid’s spine. (**A**) Stable (shaded green) and unstable (shaded red) regions of motion in the sagittal (X-Z) plane; the trajectory of the shoulder mount centroid during the experiment is shown in dark teal, (**B**) Stable (shaded green) and unstable (shaded red) regions of motion in the lateral (Y-Z) plane; the trajectory of the shoulder mount centroid during the experiment is shown in dark teal.

The cumulative range of motion for the Flexinoid shoulder mount is presented in Fig. 8. This range is directly determined by the individual motion limits of each vertebra and the coupling between sagittal and lateral movements, as shown in Figure.4 A. The limits of stable sagittal and lateral motion (Fig. 8A and B) correspond to the maximum flexion and lateral flexion of individual vertebrae (Fig. 4C and D). The limitations imposed by coupled sagittal and lateral motion are illustrated in Fig. 8B. So, The Flexinoid spine represents an engineering solution, drawing inspiration from the human spine. Its design integrates a tensegrity structure, mechanical limits, and simplified intervertebral joints to achieve a balance of stability, flexibility, and durability. These features ensure even load distribution across the spine, preventing excessive wear and maintaining structural integrity. This bio-inspired design replicates the fundamental mechanics of the human spine while simplifying the overall structure for practical application in humanoid robots.

**Fig. 9:**
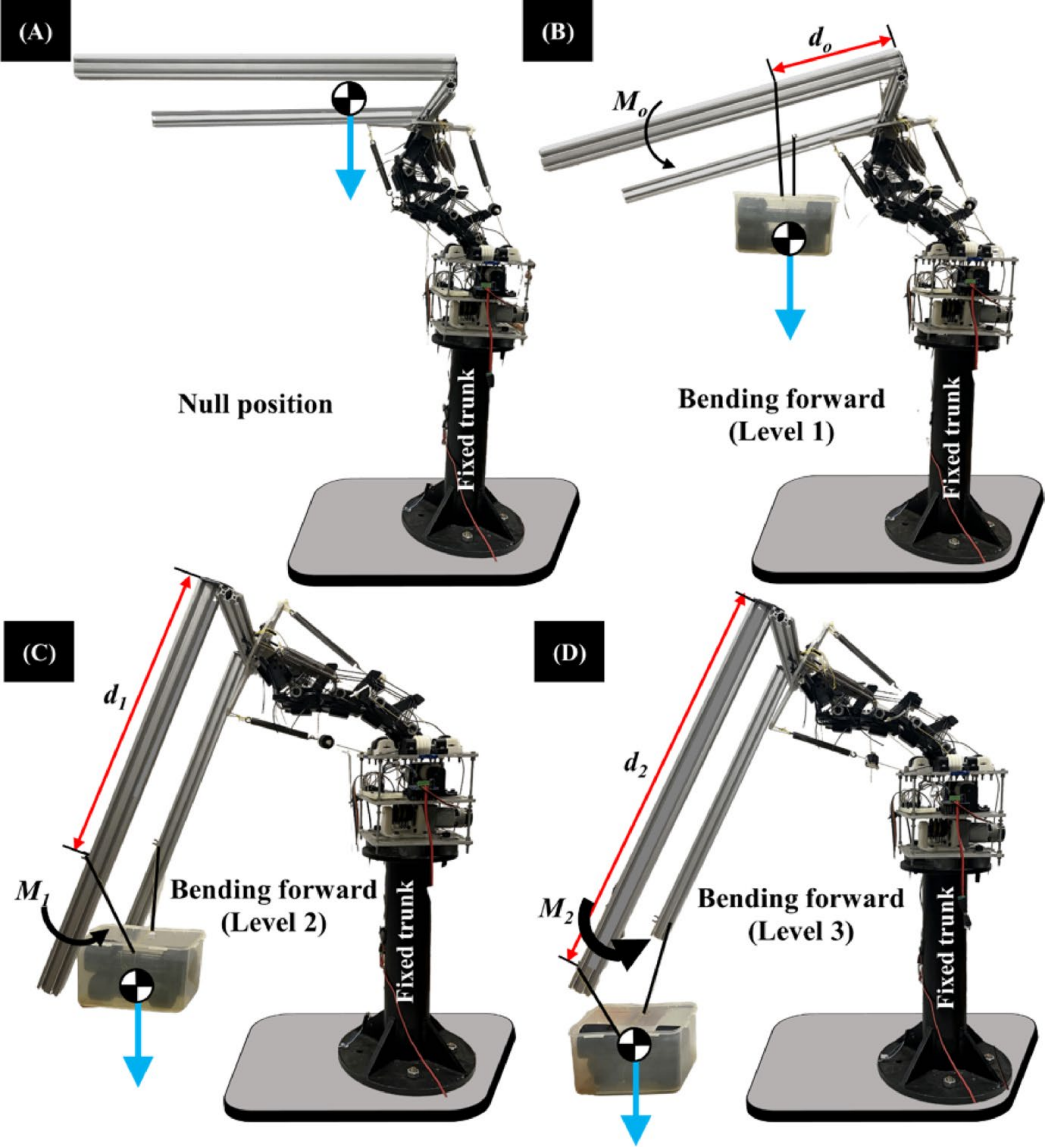
Flexinoid bending test setup and progressive flexion stages. (**A**) Null position (straight spine), (**B**) Bending forward (Level 1), (**C**) Bending forward (Level 2), (**D**) Bending forward (Level 3). Distance indicators (*d*₀, *d*₁, *d*₂) show the increased bending angle in each subsequent level, and *M₀*, *M₁*, and *M₂* represent the corresponding payload masses.

In the context of our tendon-driven system, motor back-driving refers to the phenomenon where an external load applied to the spine generates sufficient torque to overcome internal friction and mechanical losses in the gearbox, causing the unpowered motor shaft to rotate in the direction opposite to its normal driving operation. This allows the system to passively yield and move with the external load until a certain threshold, after which the motor, if powered, can then exert force to return the system towards its equilibrium position. Understanding this concept is crucial for interpreting the spine’s passive compliance and energy management. Figure 9 illustrates key qualitative observations from initial experiments conducted on the Flexinoid platform (using the experimental setup detailed in Sect. 3). These experiments revealed the spine’s capacity for passive energy storage during initial loading. As the payload was gradually increased, the spine exhibited back-drivability; however, a starting torque was required to overcome the gearbox before motor rotation. This behavior highlights the significance of the passive energy storage mechanism in the spine’s dynamic response. A series of experiments was conducted to quantitatively assess the Flexinoid spine’s performance under varying payloads (0 kg to 6 kg) in addition to the 3 kg arms weight and moment arms (25 cm, 50 cm, 75 cm) as seen in Fig. 9.

## Results and discussions

Beyond active manipulation, the Flexinoid spine also exhibits passive load-bearing capacity. As illustrated in (Fig. 7), the unpowered Flexinoid trunk can passively support a static payload of up to 15 kg, demonstrating the inherent structural integrity and load distribution capabilities provided by its tensegrity design and robust mechanical limits. This passive capacity highlights the mechanism’s ability to maintain a stable configuration under substantial static loads without requiring continuous active power.

**Fig. 10:**
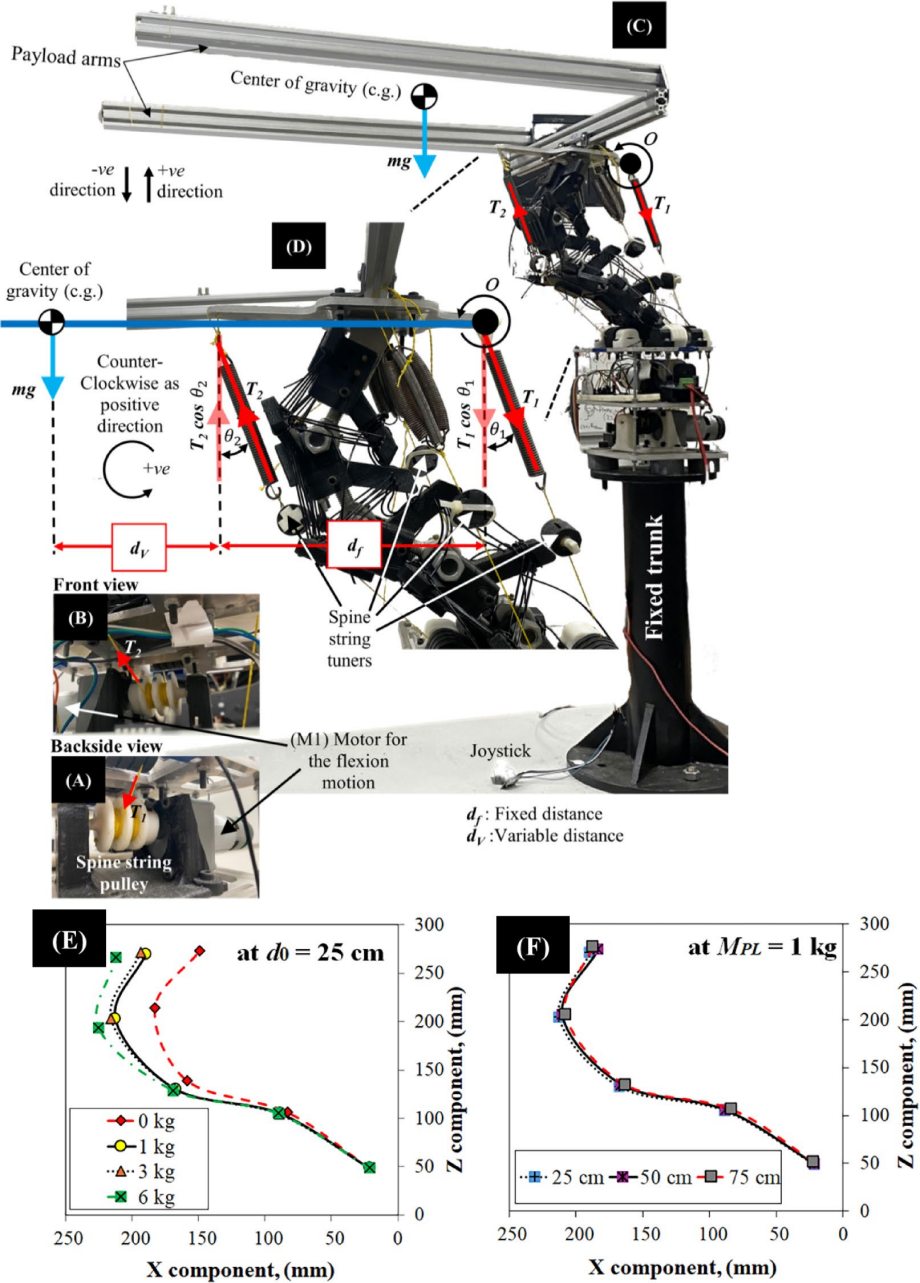

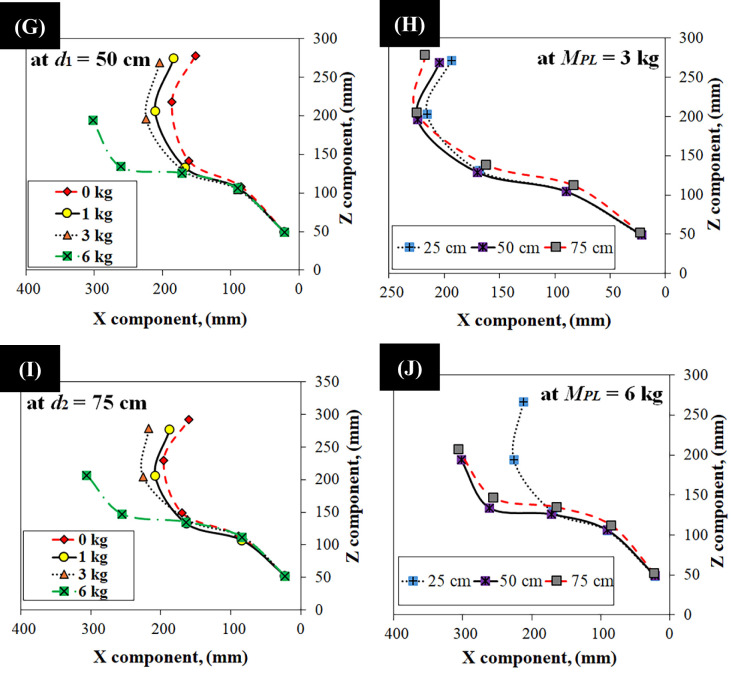
Flexinoid actuation and force analysis during sagittal plane motion. (**A**) Close-up view of the backside of the spine string pulley. Labels indicate distances (*d*_f_: fixed, *d*_v_: variable) and the (M1) motor responsible for flexion, (**B**) Close-up view of the front of the spine string tuners, (**C**) Overall experimental setup showing payload arms, center of gravity, and fixed trunk; direction of movement is indicated by arrows, (**D**) Free body diagram illustrating gravitational forces (mg) and tension forces (*T*₁, *T*₂) acting on the spine during flexion. Experimental validation for the tensions (*T*_1_, *T*_2_) versus payload mass, (**E**) Spine orientation at a distance (d₀) of 25 cm with varying payloads (0–6 kg), (**F**) Spine orientation at d₁ = 50 cm with varying payloads (0–6 kg), (**G**) Spine orientation at d₂ = 75 cm with varying payloads (0–6 kg), (**H**) Spine orientation at different distances (25–75 cm) with a payload (MPL) of 1 kg, (**I**) Spine orientation at different distances (25–75 cm) with a payload (MPL) of 3 kg, (**J**) Spine orientation at different distances (25–75 cm) with a payload (MPL) of 6 kg.

The passive energy storage mechanism observed during the initial loading phase is crucial for understanding the Flexinoid spine’s dynamic behavior. This phase involves carefully adjusted pre-tensioning in both the anterior and posterior tendons (Fig. 10A and D ), establishing the initial conditions for the quantitative analysis presented below. These initial conditions are essential for analyzing the system’s static equilibrium, which is described by the following equations of motion. The static equilibrium of the Flexinoid spine during sagittal plane motion is described by Eq. (1) through (5), derived from a force and moment balance around point *O*, the point of attachment for the actuation wire *T*₁. Equation (1), representing the summation of forces in the vertical (Z) direction, considers the gravitational forces acting on the payload arm (*mg*) and that will be varying when the payload added, as well as the vertical components of the tensions (*T*₁ and *T*₂) in the actuation wires. Solving Eq. (1) for *T*₁ yields Eq. (2), expressing *T*₁ as a function of *T*₂, the angles (*θ*₁ and *θ*₂), and the gravitational forces.

1 $$\:\sum\:{F}_{Z}=\:-{T}_{1}\:\mathrm{c}\mathrm{o}\mathrm{s}\left({\theta\:}_{1}\right)+{T}_{2}\:\mathrm{c}\mathrm{o}\mathrm{s}\left({\theta\:}_{2}\right)-mg=0$$

2 $$\:{T}_{1}=\frac{{T}_{2}\:\mathrm{c}\mathrm{o}\mathrm{s}\left({\theta\:}_{2}\right)-mg}{\mathrm{c}\mathrm{o}\mathrm{s}\left({\theta\:}_{1}\right)}$$

Equation (3) expresses the moment equilibrium around point *O*, taking into account the gravitational moments due to the payload arm. The gravitational moment is given by *mg*(*d*_f_ + *d*_v_), where *d*_f_ represents the fixed distance (23 cm) between the attachment points of actuation wires 1 and 2 on the Flexinoid’s shoulder mount, and *d*_v_ represents the variable distance to the payload. The moment caused by the tension *T*₂ in actuation wire 2 is also considered in this equation. Equation (4) is obtained by solving Eq. (3) for *T*₂.

3 $$\:\sum\:{M}_{O}=\:-{T}_{2}\mathrm{c}\mathrm{o}\mathrm{s}\left({\theta\:}_{2}\right){d}_{f}+mg\left({d}_{f}+{d}_{v}\right)=0$$

4 $$\:{T}_{2}=\frac{mg\left({d}_{f}+{d}_{v}\right)}{{d}_{f}\mathrm{c}\mathrm{o}\mathrm{s}\left({\theta\:}_{2}\right)}$$

Finally, substituting Eq. (4) into Eq. (2) yields Eq. (5), which provides a direct calculation of *T*_1_.

5 $$\:{T}_{1}=\frac{{d}_{v}mg}{{d}_{f}\mathrm{c}\mathrm{o}\mathrm{s}\left({\theta\:}_{1}\right)}$$

These equations are crucial for comprehensively understanding the static equilibrium and force balance within the Flexinoid spine, forming the foundation for the dynamic analysis described previously. Equations (1–5) provide a model for calculating the actuation wire tensions (*T*_1_ and *T*_2_), essential for predicting and controlling the Flexinoid spine’s movement and load-bearing capabilities, aligning directly with the goals of this research.

The experimental data presented in Fig. 10E and J reveal a critical threshold for initiating spine flexion: sufficient moment to overcome the gearbox. Only at distances of 50 cm and 75 cm (Fig. 10G and I) did we observe motor back-driving and resulting positive flexion; the 25 cm load (Figs. 10E) proved insufficient. Figure 10E and J further illustrate the experimentally measured spine orientation (X and Z components) under constant payloads (*M*_*PL*_ = 1, 3 and 6 kg) at these three distances. Measurements were carefully taken from each vertebra’s axis of rotation, with the fifth vertebra’s position extrapolated.

The enhanced performance metrics of the Flexinoid’s tensegrity-based spine are further supported by the results presented in Fig. 10A and D, illustrating the relationship between payload mass and actuation wire tensions (*T*₁ and *T*₂). Interestingly, the experimental results reveal an anomaly: in several cases, tension *T*_2_ exceeded tension* T*_1_. This discrepancy, not reflected in the initial static model and contrary to the observations in experimental procedures, highlights the limitations of the simplified 2D model and points to the influence of non-linear effects (due to the spine’s S-curve and tensegrity) on the system’s behavior.

This model demonstrates that *T*_1_ and *T*_2_ are determined by the payload mass, the variable distance to the payload (*d*v), and the angles *θ*_1_ and *θ*_2_ (as observed, when we used the joystick through the open-loop control). The current experimental setup lacks sensors to measure these parameters directly, representing a current limitation of this study. Future research will focus on developing closed-loop control, integrating a soft Polydimethylsiloxane (PDMS)-based strain gauge sensor with machine learning (e.g., Artificial Neural Networks (ANNs)) and Whispering Gallery Optical Modes (WGM) optical sensors^29^ to provide real-time feedback of *T*_1_, *T*_2_, *θ*₁, and *θ*₂ for enhanced precision and stability.

To quantitatively assess the Flexinoid spine’s performance metrics, we define three key parameters. Sensitivity is defined as the transfer function of the system, representing the ratio of the output (tension in Newtons, N) to the input (payload mass in kilograms, kg), thus having units of N/kg. It reflects how responsive the tension in a wire is to changes in the applied payload. The standard deviation (N) quantifies the dispersion of measured tension values around the best-fit calibration line, indicating the variability or noise in the output. Resolution (kg), representing the minimum detectable change in the input (payload), is calculated as the ratio of the standard deviation (N) to the sensitivity (N/kg). A lower resolution value indicates a system’s higher ability to discern small changes in payload, signifying greater precision in sensing the input. Therefore, resolution is directly proportional to the standard deviation and inversely proportional to sensitivity.

**Fig. 11:**
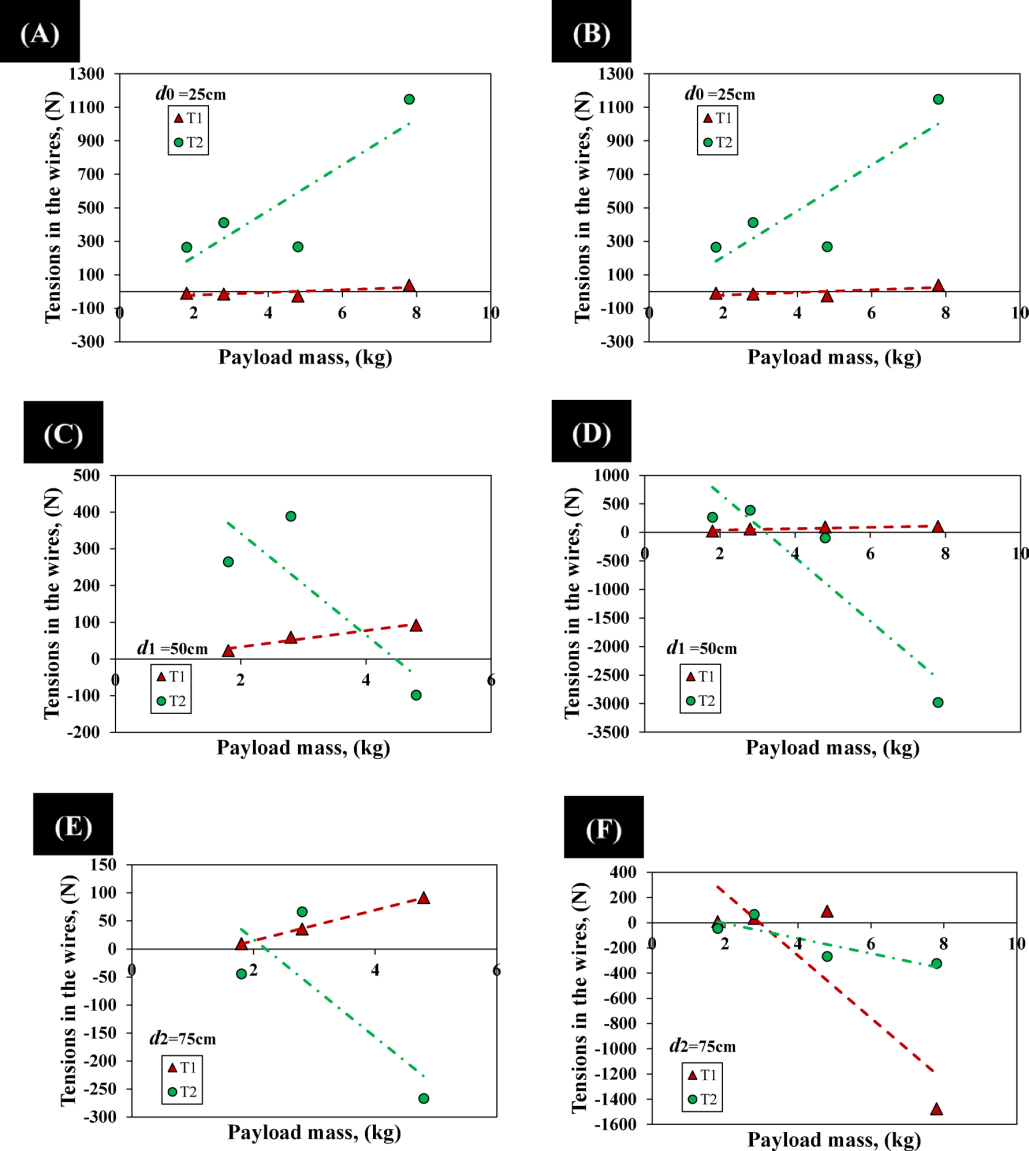
Experimental spine orientation data under varying Conditions. (**A**) at *d*₀ = 25 cm before motor back-driving, (**B**) at *d*₀ = 25 cm after motor back-driving, (C) at *d*₁ = 50 cm before motor back-driving, (**D**) at *d*₁ = 50 cm after motor back-driving, (**E**) at *d*₂ = 75 cm before motor back-driving, (**F**) at *d*_2_ = 75 cm after motor back-driving.

Experimental data, collected to validate the theoretical model (Eqs. 1–5), measure tensions *T*₁ and *T*₂ under varying payloads (0 kg to 6 kg) and moment arms (25 cm, 50 cm, and 75 cm). Figure 11A and F show six distinct curves for each tension (*T*_1_ and *T*_2_), with (Fig. 11A, C and E) representing data before motor back-driving and (Fig. 11B, D and F), showing data after back-driving (observed at 6.8 kg payload). Table 1 summarizes the performance metrics for two tension configurations, *T*₁ and *T*₂, evaluated at various distances. *T*₁ exhibited lower sensitivity but better resolution prior to motor back-driving, while *T*_2_ achieved higher sensitivity but with larger variability. Notably, after enabling motor back-driving, both configurations showed increases in sensitivity, with *T*₂ achieving the highest sensitivity of 251.60 N/kg and a resolution of 1.22 kg. These results highlight the trade-off between sensitivity and resolution depending on mechanical configuration and motor control strategies.

A crucial distinction in our experimental analysis, reflected in Table 1, pertains to the spine’s operational modes. Prior to motor back-driving, the tensegrity structure and its integrated elastic elements primarily govern the spine’s passive response, leading to a position-dependent sensitivity and resolution for *T*_1_ and *T*_2_. However, once the motor’s back-driving threshold is reached, the motor becomes actively engaged in sustaining the required tension, as the intrinsic mechanical properties of the tensegrity alone are insufficient to overcome the increasing load. In this active back-driving regime, the motor operates to maintain equilibrium regardless of the precise load location along the arm, as long as the necessary tension is generated. Consequently, the sensitivity and resolution values presented for ‘After back-driving’ in Table 1 represent the average operational characteristics of the actively engaged motor-tendon system across all tested distances, as the system transitions from a purely passive structural response to an actively compensated state.

**Table 1:** Sensitivity, standard deviation, and resolution of *T*_1_ and *T*_2_ at different distances.

Parameter	Distance	T_1_, Value	T_2_, Value
Sensitivity (N/kg)	25 cm	7.91	136.75
	50 cm	22.14	138.61
	75 cm	27.42	87.31
Average (N/kg)		19.15	120.89
Std. Deviation (N)	25 cm	16.24	188.67
	50 cm	6.27	113.59
	75 cm	0.53	1003.65
Average (N)		7.68	468.64
Resolution (kg)	Before back-driving	0.78	4.56
	After back-driving	3.70	1.22
Sensitivity (N/kg)	After back-driving	89.24	251.60

A notable observation from Table 1, and visually supported by Fig. 11 (specifically comparing 11 C/D with 11E/F), is the increase in the standard deviation of *T*₂ from 113.59 N at 50 cm to 1003.65 N at 75 cm (before back-driving). This increase in variability has profound implications for control. Given the direct proportionality between resolution and standard deviation, this surge in standard deviation translates into a higher resolution value for *T*₂ at 75 cm (4.49 kg) compared to 50 cm (0.81 kg). This indicates that at longer moment arms, the system becomes less precise in discerning small changes in payload for *T*_2_. This increased variability and diminished resolution suggest that the spine is operating in a regime where its behavior is less predictable, potentially making active control more challenging. Factors contributing to this could include increased non-linear effects at extreme deflections, enhanced coupling with unmodeled lateral motion, or amplified influence of the tensegrity network’s complex force distribution at the edges of the workspace. This highlights a critical operational limit for purely passive or open-loop control at extended moment arms, underscoring the necessity for future closed-loop control strategies.

The increase in sensitivity and decrease in resolution after motor back-driving. The initial loading phase utilizes passive energy storage in the springs before motor activation. At the 25 cm distance, minimal change is observed between pre- and post-back-driving data as seen in (Fig. 9A and D), suggesting that the threshold for motor back-driving was not reached at this shortest moment arm as was clear in (Fig. 9A).

The sensitivity increases in *T*_1_ and *T*_2_ means that the spine has have higher tendency to bear more load on the actuators wires rather than depending on the internal reconfiguration of the tensegrity structure to absorb the load. The increase of sensitivity after the motor back derived means that the system is more dependent on the actuator and the energy stored on it. The resolution decrease in *T*_1_ and *T*_2_ defines the payload after which the spine will start to depend on the actuator to compensate for the load. In other words, resolution defines the limit at which the system starts to abandon flexibility (shock absorption feature) and starts to depend on the actuator to respond with stiff reaction. Based on that, the novelty of the proposed design presented in this paper lies in the integration of the mechanical hardware structure with the actuation system, enhancing the payload capacity.

The observed improvements in locomotion performance can be attributed to several key features of the Flexinoid spine design. The inherent compliance of the TPU cables, combined with the tensegrity structure’s inherent stability, allows for efficient load distribution and effective shock absorption. This reduces the strain on individual components and minimizes energy loss due to posture correction, leading to the observed improvement in energy efficiency. The tensegrity structure’s ability to maintain its structural integrity while providing flexibility passively with a wide range of motion is crucial for achieving the enhanced dexterity. The modular design enables easier assembly, maintenance, and potential future customization, enhancing the system’s overall robustness.

Our tensegrity-based spine design offers advantages over existing designs. Traditional rigid spines exhibit limited flexibility and high energy consumption relative to its peers^30^. Multi-actuator spine designs can provide greater flexibility^20,21^ but are complex, expensive, and require control algorithms. Continuum robots also offer flexibility^22,23^, but face challenges in terms of control and load-bearing capacity. Our design provides a balance of flexibility, stability, load-bearing capacity, and energy efficiency, as evidenced by the quantitative improvements observed in our experiments.

The experimental data revealed an interesting anomaly: in some cases, the tension in wire *T*₂ exceeded the tension in wire *T*₁. This observation is not predicted by the initial static model and contradicts qualitative observations from the experimental procedures. This discrepancy can be attributed to the limitations of our simplified 2D model, which neglects certain factors, such as non-linear effects due to the spine’s S-shaped curve and the influence of tensegrity on the system’s behavior, as well as the influence of lateral flexion.

Future research should focus on developing more models that address the observed anomaly, ideally incorporating a 3D model and dynamic simulation. Further experimental work will involve implementing a closed-loop control system, utilizing a novel sensor system based on soft PDMS strain gauges combined with ultra-sensitive WGM sensors to provide real-time feedback of wire tensions and joint angles^29^. This will enable optimization of the actuation strategy and further enhancement of the spine’s performance.

## Conclusion

In conclusion, this study presents a novel bio-inspired, tensegrity-based flexible spine mechanism, offering a transformative alternative to conventional rigid-trunk designs for humanoid robots. Through a comprehensive design methodology and experimental validation on the Flexinoid humanoid platform, we have demonstrated the following key findings; the spine mechanism provides an enhanced range of motion, achieving flexion from − 30° to 65° and lateral bending of ± 30°. Its intrinsic structural properties enable it to passively support substantial static loads, up to 15 kg, and to absorb impulsive shocks, contributing to the overall stability of the trunk. Furthermore, the system improved sensitivity and resolution in tension measurement following motor back-driving, validating the effectiveness of its integrated passive energy storage mechanism during initial load accommodation. These properties collectively reduce the active burden on actuators required for trunk manipulation and highlight the spine’s inherent compliant and load-redistributing capabilities. This research advances the field of compliant mechanism design by offering a robust, adaptable, and energy-efficient solution that leverages the unique balance of compliance, intrinsic stability, and modularity inherent in tensegrity structures. It lays a foundational framework for future humanoid systems capable of more dynamic, responsive, and robust interactions. Future work will focus on integrating advanced sensing and control strategies, such as soft (PDMS)-based strain gauges combined with ultra-sensitive WGM optical sensors, to enable real-time feedback and closed-loop control. This will allow for a more comprehensive 3D dynamic modeling, further refinement of the design, and quantitative validation of full system-level dynamic stability and energy efficiency across diverse applications.

## Data Availability

All original data generated and/or analysed in the current study are included in the article, further inquiries can be directed to the corresponding author.
